# APACHE III outcome prediction in patients admitted to the intensive care unit after liver transplantation: a retrospective cohort study

**DOI:** 10.1186/1471-2482-9-11

**Published:** 2009-07-29

**Authors:** Mark T Keegan, Bhargavi Gali, James Y Findlay, Julie K Heimbach, David J Plevak, Bekele Afessa

**Affiliations:** 1 Division of Critical Care, Department of Anesthesiology, Mayo Clinic, Rochester, MN, USA; 2 Division of Transplantation Surgery, Department of Surgery, Mayo Clinic, Rochester, MN, USA; 3 Department of Anesthesiology, Mayo Clinic, Rochester, MN, USA; 4 Division of Pulmonary and Critical Care Medicine, Department of Internal Medicine, Mayo Clinic, Rochester, MN, USA

## Abstract

**Background:**

The Acute Physiology and Chronic Health Evaluation (APACHE) III prognostic system has not been previously validated in patients admitted to the intensive care unit (ICU) after orthotopic liver transplantation (OLT). We hypothesized that APACHE III would perform satisfactorily in patients after OLT

**Methods:**

A retrospective cohort study was performed. Patients admitted to the ICU after OLT between July 1996 and May 2008 were identified. Data were abstracted from the institutional APACHE III and liver transplantation databases and individual patient medical records. Standardized mortality ratios (with 95% confidence intervals) were calculated by dividing the observed mortality rates by the rates predicted by APACHE III. The area under the receiver operating characteristic curve (AUC) and the Hosmer-Lemeshow C statistic were used to assess, respectively, discrimination and calibration of APACHE III.

**Results:**

APACHE III data were available for 918 admissions after OLT. Mean (standard deviation [SD]) APACHE III (APIII) and Acute Physiology (APS) scores on the day of transplant were 60.5 (25.8) and 50.8 (23.6), respectively. Mean (SD) predicted ICU and hospital mortality rates were 7.3% (15.4) and 10.6% (18.9), respectively. The observed ICU and hospital mortality rates were 1.1% and 3.4%, respectively. The standardized ICU and hospital mortality ratios with their 95% C.I. were 0.15 (0.07 to 0.27) and 0.32 (0.22 to 0.45), respectively.

There were statistically significant differences in APS, APIII, predicted ICU and predicted hospital mortality between survivors and non-survivors. In predicting mortality, the AUC of APACHE III prediction of hospital death was 0.65 (95% CI, 0.62 to 0.68). The Hosmer-Lemeshow C statistic was 5.288 with a p value of 0.871 (10 degrees of freedom).

**Conclusion:**

APACHE III discriminates poorly between survivors and non-survivors of patients admitted to the ICU after OLT. Though APACHE III has been shown to be valid in heterogenous populations and in certain groups of patients with specific diagnoses, it should be used with caution – if used at all – in recipients of liver transplantation.

## Background

Intensive care units (ICUs) have played a vital role in the practice of orthotopic liver transplantation (OLT). Although there have been moves towards avoidance of ICU admission after transplantation, most patients still spend part of their post-operative course in the ICU.[1,2]

A variety of scoring systems have been used to quantify the severity of illness of patients admitted to the ICU and to predict their chances of survival to ICU and hospital discharge.[3] Such prognostic scoring systems include the Simplified Acute Physiology Score (SAPS), the Mortality Probability Model (MPM) and the Acute Physiology and Chronic Health Evaluation (APACHE) scoring system. [4-6] APACHE was introduced in the early 1980s and while minor modifications have been made over the years, only 3 major revisions have occurred. [6-8] APACHE III, originally published in 1991, has been used in a significant number of ICUs, especially in the United States, for the past 15 years.[8]

Mortality prediction models are likely to under- or over-estimate mortality in selected patient subpopulations which were not well-represented in the original cohort. For example, studies of kidney transplant recipients and patients with malignancies admitted to the ICU have shown the inability of the APACHE and SAPS scoring systems to accurately predict mortality in these patient groups. [9-12]

Of the cohort of 17,440 patients used for the development and validation of the original APACHE III prognostic model only 40 patients were admitted to the ICU after OLT.[8] Between 4000 and 5000 OLTs are performed every year in the United States. While an older version of the APACHE scoring system, (APACHE II) has been studied in patients after OLT, the predictive ability of the APACHE III and IV systems has not been examined.[10,13-15]

The aim of our study was to validate the APACHE III scoring system in a large cohort of patients admitted to the ICU of a tertiary referral center immediately after OLT. In addition we aimed to evaluate the impact of the addition of other variables (e.g. the Model for End-Stage Liver Disease, MELD) on the predictive ability of APACHE. Prognostic systems have been used to justify the development of progressive care units by identification of a group of ICU patients at low risk for mortality. [16-18] Such systems may also provide objective assessment for the development of ICU discharge criteria and may identify those patients likely to require ICU readmission.[19] Both of these issues were of interest to our institutional leadership at the time of the study.

The liver transplant program at Mayo Clinic Rochester began in 1985. In the immediate post-operative period, all patients that have undergone OLT (except pediatric patients and patients who have undergone combined liver and heart transplantation) are cared for in the multidisciplinary ICU at Rochester Methodist Hospital, one of the Mayo Clinic-affiliated hospitals in Rochester. This unit has expanded from the original 12 beds, to 17 beds in March 2000, and to 21 beds in April 2008. It is staffed by a multidisciplinary critical care team who provide care to solid organ and bone marrow transplant recipients, and hematology and oncology, general surgery and orthopedic patients. Acute ICU management of patients after OLT is provided by senior anesthesia residents or critical care fellows working under the supervision of board certified intensivists. Patients are managed in conjunction with the liver transplantation service which consists of both transplant surgeons and hepatologists. The APACHE III scoring system has been employed in a prospective fashion for all patients in this ICU since July 1996. Although the APACHE IV equation for the prediction of hospital mortality is in the public domain, other predictive equations are not, and our institution has chosen not to upgrade to the commercially-available APACHE IV package at this time.

## Methods

A retrospective cohort study was performed after Institutional Review Board approval and the granting of a waiver of informed consent. From the institutional liver transplant databases adult patients who underwent OLT between July 1996 and May 2008 were identified. For each of the identified patients the institutional APACHE III database was searched, using the software provided by Cerner Corporation (Kansas City, Missouri), and APACHE III data were abstracted. Patients who did not authorize a review of their medical records for research and pediatric patients were excluded. Patients who died in the operating room or soon after arrival to the ICU were also excluded as only patients who spend more than four hours in the ICU generate APACHE data. Data were collected for the day of ICU admission after liver transplantation only. Data for second or subsequent ICU admissions were not used.

The entry of all laboratory values used for the APACHE III scoring was computerized using software that interfaces with the laboratory. Vital signs, Glasgow Coma Scale scores and urine output were abstracted by the bedside nurses according to a formalized protocol and entered into the computer by trained specialists. The nurses received training, an instruction manual and initial supervision. Audit of the collected data for missing and discrepant admission, physiologic and outcome values was performed by experienced clinical ICU nurses. To successfully pass the audit, criteria were set including: at least 90% agreement on admission variables overall, 100% agreement on admission and discharge dates, a minimum of 80% agreement in admission diagnosis, admission and discharge times, chronic health items, readmission status, surgical status, active treatment status and at least 85% agreement on overall physiology variables. Use of the APACHE III database at our institution has been previously described.[20]

The data abstracted included age, gender, acute physiology score (APS), APACHE III score (APIII), APACHE III-predicted ICU and hospital mortality, and predicted length of ICU and hospital stay. The APS and APACHE III scores for each patient were calculated as described by Knaus and colleagues.[8] Additionally, patients' actual lengths of ICU and hospital stay, and ICU and hospital discharge status (survivor versus non-survivor) and discharge location were recorded.

In addition to variables abstracted from the APACHE database, the institutional anesthesia and liver transplant databases were searched and data regarding duration of anesthesia and surgery and intraoperative administration of packed red blood cells (PRBCs) were recorded for each patient. For patients transplanted after February 2002 (when the MELD score was adopted by the United Network for Organ Sharing as the basis for allocation of donor organs), the MELD score at the time of transplantation was abstracted.

Descriptive data are summarized as mean (standard deviation, SD), median (interquartile range, IQR) or percentage. A chi-square analysis was used to compare categorical variables and Student's t test and rank sum tests were used to compare continuous variables. Patients with missing data were excluded from the analyses involving the missing data. Statistical tests were two-tailed and tests were considered statistically significant with P < 0.05.

Standardized mortality ratios (SMRs) were calculated by dividing the observed rates by the rates predicted by APACHE III. The 95% confidence intervals (CI) were calculated for each of the standardized mortality ratios. Discrimination of a prognostic model is the ability of the model to distinguish between survivors and non-survivors. The discrimination of the APACHE III- predicted mortality for the prediction of in-hospital mortality was analyzed by calculating the area under the receiver operating characteristic curve (AUC).[21] An AUC of > 0.9 was considered to be outstanding, greater than 0.8 to 0.9 excellent, 0.7 to 0.8 acceptable, and less than 0.7 was considered poor. Calibration of a model is the degree of agreement between predicted mortality and actual mortality. The Hosmer-Lemeshow C statistic was used to determine the calibration of the model. A model with good calibration should have a Hosmer-Lemeshow statistic close to the degrees of freedom, which is equal to the number of categories minus 2, and a P-value > 0.05.[22] The Brier score was used as an overall assessment of the model's performance, with a lower score indicating better performance.[23]

Data analyses were performed using SPSS 11.5 (SPSS Inc., Chicago, IL) and MedCalc Version 9.1 (MedCalc Software, Mariakerke, Belgium.)

## Results

APACHE III data were available for 918 admissions after OLT. The baseline characteristics of the subjects in the study are shown in Table 1. The patients' mean age was 51.5 years (S.D. 10.8). Eighty-five percent were Caucasian and 579 (63.0%) were male. The mean APIII on the day of transplant was 60.5 (SD 25.8) and the mean APS was 50.8 (23.6). Median (IQR) lengths of ICU and hospital stay were 1.2 (0.9, 2.0) and 10.4 (8.3, 18.9) days, respectively.

**Table 1:** Characteristics of 918 patients who underwent orthotopic liver transplantation between 1996 and 2008

Age, yrs, mean (S.D)	51.5 (10.8)
Male	579
Female	339
MELD*, mean (S.D)	25.0 (7.7)
Caucasian	784 (85.5%)
Total anesthesia time (mins.), median (IQR)	384 (326, 449)
Total surgical time (mins.), median (IQR)	297 (240, 361)
Total anhepatic time (mins.), median (IQR)	75 (63, 89)
Intraop PRBCs (units), median (IQR)	5.0 (3.0, 8.0)
APS, mean (S.D.)	50.8 (23.6)
APIII, mean (S.D.)	60.5 (25.8)
Pred. number of ICU deaths (% of total)	67 (7.3%)
Actual number of ICU deaths (% of total)	10 (1.1%)
Pred. ICU LOS, median days (IQR)	4.4 (3.4, 5.7)
Actual ICU LOS, median days (IQR)	1.2 (0.9, 2.0)
Pred. number Hosp. deaths (% of total)	97 (10.6%)
Actual number Hosp. deaths (% of total)	31 (3.4%)
Pred. Hosp LOS days, median (IQR)	15.9 (13.1, 22.6)
Actual Hosp. LOS days, median (IQR)	10.4 (8.3, 18.9)
Pred Vent days, median (IQR)	3.5 (2.3, 4.1)
Survival to ICU discharge, number (% of total)	908 (98.9%)
Survival to hospital discharge, number (% of total)	887 (96.6%)

*MELD data are for 514 patients

Pred. = Predicted

LOS = Length of stay

S.D. = Standard deviation

IQR = Interquartile range

Hosp. = Hospital

ICU = Intensive Care Unit

LOS = Length of stay

APS = Acute Physiology Score

APII = Acute Physiology and Chronic Health Evaluation III

Intra-op PRBCs = Intraoperative packed red blood cells

MELD = Model for end-stage liver disease

Mins. = minutes

Yrs = years

Mean (SD) predicted ICU and hospital mortality rates were 7.3% (15.4) and 10.6% (18.9), respectively. The observed ICU mortality rate was 1.1% (10 deaths of 918 patients admitted to the ICU after transplantation.) The hospital mortality rate was 3.4% (31 deaths in 918 patients.) Thus, 21 deaths occurred in patients after discharge from the ICU. These included patients in whom support was withdrawn on the surgical floor or non-ICU ventilator dependency unit, in whom a decision had been made not to readmit to the ICU, or in whom a sudden decompensation (e.g. cardiac arrest) occurred which led to the patient's death before transfer back to the ICU. The standardized ICU and hospital mortality ratios with their 95% C.I. were 0.15 (0.07 to 0.27) and 0.32 (0.22 to 0.45), respectively.

MELD data were available for 514 patients. The mean MELD score on the day of transplantation was 25.0 (SD 7.7). There was no difference in mean MELD score between survivors and non-survivors. The median number of PRBCs transfused was 4.0 and this was greater in non-survivors than survivors (P < 0.01).

Among the 843 survivors to hospital discharge, 875 (91.8%) were discharged to home (as opposed to another hospital or rehabilitation facility.) There were no significant differences in gender (P = 0.57), race (P = 0.73) or age (P= 0.51) between survivors and non-survivors. There were statistically significant differences in APS, APIII, predicted ICU and predicted hospital mortality between survivors and non-survivors. In addition, the predicted and actual ICU and hospital lengths of stay were significantly different between survivors and non-survivors. Comparisons of survivors to hospital discharge and non-survivors are in Table 2.

**Table 2:** Comparison of survivors to hospital discharge and non-survivors

	Survivor	Non-survivor	P-value
	N = 887	N = 31	
Age (yrs)	51.5 (10.8)	52.8 (11.0)	0.51
Male	561	18	
Female	326	13	0.57
MELD*	25.0 (7.7)	25.6 (8.0)	0.78
Caucasian	757	27	0.73
Total anesthesia time (mins.)	384 (325, 447)	424 (351, 494)	0.05
Total surgical time (mins.)	295 (239, 361)	329 (254, 403)	0.09
Total anhepatic time (mins.)	75 (63, 89)	77 (66, 95)	0.7
Intra-op PRBCs (units)	4.0 (2.0, 7.0)	9.5 (4.3, 16.8)	< 0.01
APS	50.3 (23.1)	66.6 (31.9)	< 0.01
APIII	59.9 (25.2)	79.0 (34.5)	< 0.01
Pred. ICU death %, (S.D.)	7.0 (14.8)	17.6 (25.9)	< 0.01
Pred. ICU LOS days, median (IQR)	4.4 (3.4, 5.6)	5.6 (4.2, 6.7)	< 0.01
Actual ICU LOS days, median (IQR)	1.2 (0.9, 2.0)	2.8 (1.1, 8.8)	< 0.01
Pred Hosp death %, (S.D.)	10.2 (18.4)	22.2 (28.5)	< 0.01
Pred Hosp LOS days, median (IQR)	15.8 (13.1, 22.4)	20.0 (15.3, 23.9)	0.03
Actual Hosp LOS days, median (IQR)	10.3 (8.3, 18.1)	21.2 (7.9, 37.2)	0.02
Pred ventilator days, median (IQR)	3.5 (2.3, 4.1)	3.6 (2.9, 4.3)	0.16

Values are means (standard deviation) or medians (interquartile range)

*MELD data are for 514 patients

APS = Acute Physiology Score

APII = Acute Physiology and Chronic Health Evaluation III

Pred. = Predicted

Hosp. = Hospital

ICU = Intensive Care Unit

LOS = Length of stay

Intra-op PRBCs = Intraoperative packed red blood cells

MELD = Model for end-stage liver disease

Mins. = minutes

Yrs = years

**Figure 1:**
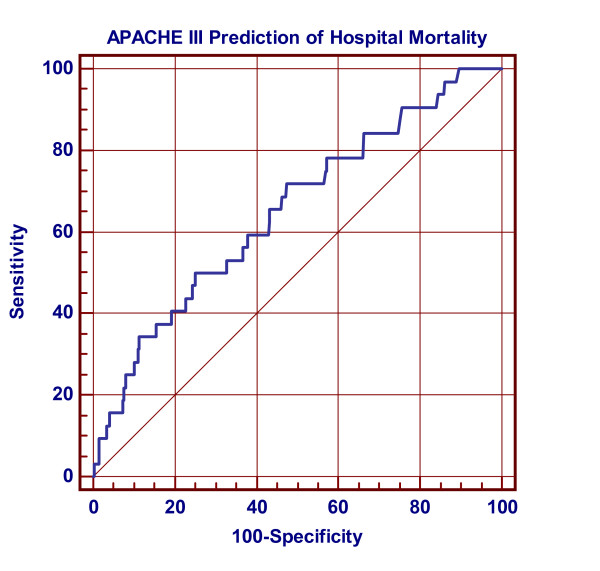
**Receiver operator characteristic plot of hospital mortality prediction using the APACHE III-prediction of hospital death**. AUC of APACHE III prediction of hospital death was 0.65 (95% CI, 0.62 to 0.68). Diagonal line denotes AUC = 0.5.

In predicting mortality, the AUC of APACHE III prediction of hospital death was 0.65 (95% CI, 0.62 to 0.68). The receiver operator characteristic curve is shown in Figure 1. The Hosmer-Lemeshow C statistic was 5.288 with a P-value of 0.871 (10 degrees of freedom). The observed and APACHE III-predicted numbers of hospital survivors and non-survivors according to deciles of risk are given in Table 3. The Brier score was 0.065.

**Table 3:** Observed and APACHE III-predicted number of survivors and non-survivors to hospital discharge according to deciles of risk

	Survivors		Non-survivors	
Decile of risk	Observed	Expected	Observed	Expected
1	94	91.7	0	2.3
2	87	87.7	3	2.3
3	91	90/6	2	2.4
4	90	88.7	1	2.3
5	90	89.6	2	2.4
6	89	90.5	4	2.5
7	89	89.5	3	2.5
8	88	89.3	4	2.7
9	88	88.5	4	3.5
10	81	80.9	8	8.1

The Hosmer-Lemeshow C statistic was 5.288 with a p value of 0.871 (10 degrees of freedom).

In an effort to identify a model with better discriminatory power while maintaining satisfactory calibration, further analyses were performed and a series of multiple logistic regression models were developed. In addition to inclusion of the APACHE III prediction of hospital mortality, these regression models included combinations of following variables: duration of anesthesia, duration of surgery, anhepatic time, number of PRBCs transfused, and MELD. The model that included APACHE III-prediction of mortality and MELD had an AUC of 0.67. A model that included APACHE III-prediction of mortality, MELD, anesthesia time and the number of PRBCs transfused provided a slightly higher AUC at 0.74, though this model still had only barely acceptable discriminatory ability.

## Discussion

The results from our study demonstrate that the APACHE III prognostic scoring system poorly discriminates between survivors and non-survivors in a large group of patients admitted to the ICU after OLT. The AUC for APACHE III-calculated prediction of death before hospital discharge was only 0.65, which is not acceptable for discrimination. The SMRs were very low (0.15 and 0.32 for the ICU and hospital SMR, respectively) suggesting that the model overestimated the mortality for a given severity of illness. Although the Hosmer-Lemeshow statistic was 5.288 with a p value of 0.871 indicating good calibration and the Brier score was low, the poor discriminatory ability means that the model is not robust enough to be used for individual mortality prediction or for cohort prognosis.

In physiologic scoring systems, the accuracy of prediction is not perfect and it is unreasonable to expect a very high AUC. The best predictive models have AUCs that approach 0.9. However, our data demonstrate that the APACHE III system, when used for patients after OLT, falls significantly below this desired level, and does not meet criteria to even be deemed "acceptable" as a prognostic tool.

A variety of factors may have contributed to the poor performance of APACHE III in this population. Errors in data acquisition, interpretation, and application of the APACHE III equations may have been responsible. However, our experience with a large and heterogenous group of patients at our institution suggests that we have acquired data and implemented APACHE III properly.[20] Mortality after OLT may result from the sequelae of rejection, which (except for primary non-function) are unlikely to be seen during a patient's post-OLT ICU stay. Similarly, opportunistic infection arising in the setting of immunosuppression may lead to patient demise, though is unlikely to manifest on the first ICU day. Intraoperative resuscitation and therapy will "normalize" many physiologic derangements. As laboratory and physiologic data acquired before admission to the ICU are not included in calculation of the APS or APIII, ICU calculations may underestimate the severity of illness of an OLT recipient. Finally – and most likely the primary reason for failure of the prognostic model – the proprietary equations from which predictions are calculated were based on a development cohort with few patients after OLT. The inherent benefit of transplantation is that it has the potential to completely reverse a patient's deteriorating physiology, thus modifying the risk of mortality, and this benefit does not appear to be adequately accounted for in APACHE.

Customization of prognostic scoring systems has been attempted, because mortality predictions are often not accurate in populations other than those in which the systems were developed because of differences in case-mix.[24,25] Angus and colleagues customized APACHE II (not III) for prediction of mortality in OLT recipients. However, even their customized model had poor discrimination (AUC 0.68 to 0.72).[15] Another attempt at customization of APACHE II for use in OLT recipients by Arabi and colleagues resulted in an improvement of discrimination and calibration.[13] However, this customization has been criticized because of the use of a highly heterogenous database and a sample size too small to use the Hosmer-Lemeshow goodness of fit test appropriately.[26] Similar to the experiences of other investigators with APACHE II, customization did not significantly improve the performance of APACHE III in our study. It is not surprising that customization of the model by inclusion of the MELD score did not result in a significant improvement in discriminatory ability. Most of the variables upon which MELD is based are already included as part of the APACHE prediction model. (Bilirubin, creatinine, and the presence of renal failure or cirrhosis are included in APACHE, though INR is not.) However, even the addition of other variables did not provide a model with good or excellent discriminatory ability.

There are number of limitations to our study. Patients were cared for at a single center and may not be representative of other ICUs in other medical centers. However, the validation of APACHE in a group of patients at a single center may more accurately reflect the performance of the model without the confounding influence of standards of care at different institutions. Thus, we believe that APACHE III is unlikely to perform better in OLT recipients elsewhere. Similar to other investigators using earlier models of APACHE, the calibration of the model was good. This is interesting, as the calibration of scoring systems usually declines over time.[3] However, the low number of deaths limits the power of the calibration analysis. The population studied was devoid of racial diversity as it comprised a large number of Caucasians. While this reflects the referral practice of our institution, it may limit the generalizability of our findings. There was a variety of etiologies for liver failure in our patient population and APACHE may perform better in certain disease states, though we did not have a sufficient patient population to test this hypothesis. Furthermore, heterogeneity of the populations in the pre- and post- MELD era may also be a confounding factor. The exclusion of patients who died in the operating room or within four hours of ICU admission because they did not generate APACHE data may have further limited our analyses.

## Conclusion

The APACHE III scoring system discriminates poorly between survivors and non-survivors of patients admitted to the ICU after OLT. Customization of APACHE III, including the addition of MELD, did not result in acceptable discrimination of the model. Though APACHE III has been shown to be valid in heterogenous populations and in certain groups of patients with specific diagnoses, it should be used with caution – if used at all – in recipients of liver transplantation.

## Abbreviations

ICU: Intensive Care Unit; APACHE: Acute Physiology and Chronic Health Evaluation; SAPS: Simplified Acute Physiology Score; MPM: Mortality Probability Model; OLT: Orthotopic Liver Transplantation; MELD: Model for End-Stage Liver Disease; SMR: Standardized Mortality Ratio; SD: Standard Deviation; AUC: Area Under the Receiver Operating Characteristic Curve

## Competing interests

The authors declare that they have no competing interests.

Funded was provided solely through institutional funds

## Authors' contributions

MTK designed the study, acquired and analyzed the data and wrote the manuscript

BG, JYF, DJP participated in study design, interpretation of data and critically revised the manuscript for important intellectual content

JKH acquired some of the data and participated in interpretation of data and critical revision of the manuscript for important intellectual content

BA participated in study design and interpretation of data and critically revised the manuscript for important intellectual content

All authors approved the final manuscript

## Authors' Information

Mark T. Keegan, MB, MRCPI: Associate Professor of Anesthesiology

Bhargavi Gali, M.D. Assistant Professor of Anesthesiology

James Y. Findlay, MB ChB FRCA Assistant Professor of Anesthesiology

Julie K. Heimbach, M.D. Assistant Professor of Surgery

David J. Plevak, M.D. Professor of Anesthesiology

Bekele Afessa, M.D. Associate Professor of Medicine

## Pre-publication history

The pre-publication history for this paper can be accessed here:


